# Double Salts and Racemate in Mefloquine–Ibuprofen and Mefloquine–Ketoprofen Systems. A Structural and Thermochemical Analysis

**DOI:** 10.1002/chir.70088

**Published:** 2026-02-13

**Authors:** Juliana B. Martins, João V. Segatto, Juan C. Tenorio, Paulo S. Carvalho

**Affiliations:** ^1^ Institute of Physics Federal University of Mato Grosso do Sul Campo Grande Mato Grosso do Sul Brazil; ^2^ Physics Institute Universidade Federal do Rio de Janeiro Rio de Janeiro Rio de Janeiro Brazil

## Abstract

The formation of salts remains a highly sought‐after yet unpredictable strategy for resolving chiral drugs. Using a drug–drug approach, we have investigated the crystallization behavior of Mefloquine (Mf), a racemic antimalarial drug, in combination with enantiopure (S)‐ibuprofen and (S)‐ketoprofen, as well as their racemic forms. Double salts and racemates have been obtained and characterized by single‐crystal and powder X‐ray diffraction, Hirshfeld surface analysis, thermal analysis (DSC and TGA), and solubility measurements. Structural analysis reveals that the formation of double salts often follows a pattern similar to the packing and supramolecular motifs found in the corresponding racemates. For the ketoprofen systems, the double salt and racemate have been found to be isostructural and isomorphic, whereas the ibuprofen systems display different architectures despite similar unit cells. Both double salts exhibited thermal stability and solubility profiles comparable to their racemic counterparts. On the other hand, the racemate with ketoprofen and the double salt with ibuprofen are the most soluble structures, suggesting that they are more stable systems than their counterparts. These findings support the view that double salt formation in such systems results from structural mimicry of racemates, emphasizing the challenges of predictable enantiomeric resolution and the importance of understanding structure–property relationships in chiral crystallization.

## Graphical abstract

(S)‐ibuprofen or ketoprofen were used to resolve mefloquine, forming double salts structurally similar to the racemic forms.

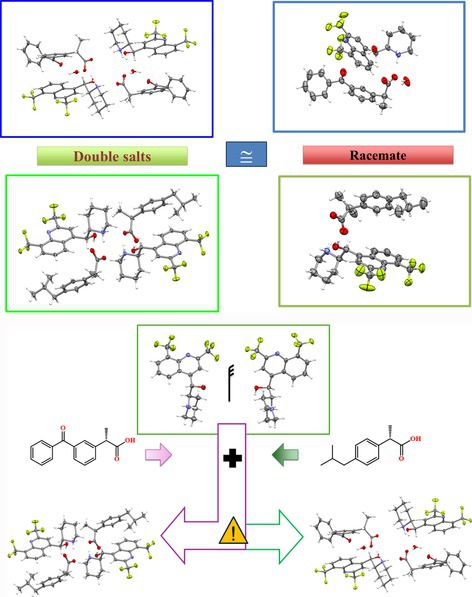

## 1 Introduction

Due to safety considerations and the goal of improving therapeutic effectiveness, there is an increasing trend toward enantiomerically pure drugs rather than racemic mixtures in pharmaceutical formulations [1, 2, 3, 4]. This trend is significant, as over 50% of drugs currently on the market are chiral [5]. Notably, enantiomeric separation can be achieved through several approaches [6], among which crystallization‐based methods stand out due to their versatility, scalability, cost‐effectiveness, and operational efficiency [7, 8, 9].

Forming diastereomeric salts or cocrystals is among the most common strategies for separating enantiomers [10, 11]. This method exploits the distinct physical properties of diastereomers to facilitate their separation. However, while the initial synthesis of these salts may be straightforward, the subsequent crystallization process introduces additional complexity in enantiomeric purification. Interaction between a racemic compound and a resolving agent can lead to a variety of solid phases—including solid solutions, double salts, and polymorphs—all of which can compromise resolution efficiency [12]. This inherent unpredictability in solid‐state chemistry makes selecting an appropriate resolving agent a significant challenge, as unwanted phase transitions often hinder purification success.

Significant advances in engineered multicomponent crystals have greatly enhanced the chiral resolution of active pharmaceutical ingredients (APIs) [6, 13, 14]. Understanding of the diverse systems resulting from resolution processes [11, 15, 16] can provide essential guidance for designing and interpreting chiral discrimination mechanisms. Among these phases, double salts [15] are often regarded as an undesired outcome. However, investigating the underlying reasons for their formation [17, 18]—as well as the structure–property relations between these double salts and their racemic counterparts—represents a promising approach for optimizing resolution strategies. Despite their importance, the literature on double salts involving API enantiomers remains limited. The formation of diastereomeric double salts is not uncommon and has frequently been associated with limited enantiomeric recognition during crystallization. Currently, there are no reliable rules based on structural similarities or differences between diastereomeric salts that allow for the prediction of double salt formation. For example, attempts to resolve specific racemic N‐(3,5‐dinitrobenzoyl)amino acids via fractional crystallization of brucinium diastereomeric salts have instead led to the formation of diastereomeric double salts [18], illustrating the unpredictable nature of these systems.

**SCHEME 1:**
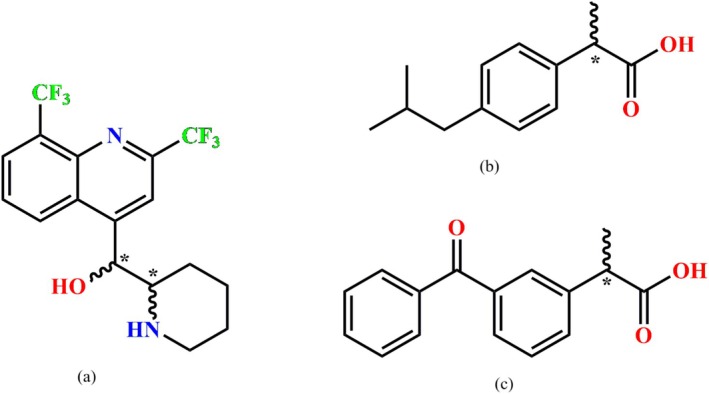
Molecular structure of (a) Mefloquine, (b) Ibuprofen, and (c) Ketoprofen. The (*) indicates the asymmetric carbon.

Mefloquine [19, 20, 21] (Mf, Scheme 1) is an antimalarial agent widely used since 1980 [21].

The molecule has two asymmetric carbons and is administered pharmaceutically as a racemic mixture of the two enantiomers. The (+)‐Mf enantiomer is more effective against malaria than the (−)‐Mf enantiomer, which is associated with undesirable central nervous system side effects [22, 23]. Despite its lower efficacy, the drug is administered pharmaceutically as a hydrochloride racemic salt (±‐Mf HCl), which is classified as a Class IV drug, possibly due to the existence of several polymorphic phases [24, 25]. Several crystal structures of mefloquine double salts with propionate and succinate anion‐derivatives have recently been reported [26], indicating that identifying appropriate conditions and resolving agents remains necessary for enantiomeric resolution of this API. Herein, a drug‐to‐drug strategy for multicomponent crystals was studied using enantiopure and racemic Nonsteroidal Anti‐inflammatory Drugs (NSAIDs) such as Ketoprofen (Kt) and Ibuprofen (Ib). These structures were analyzed using single‐crystal and powder X‐ray diffraction, thermal analysis methods including DSC and TGA, and solubility measurements. Analyzing the double‐drug salt formed by Mf with NSAIDs, alongside its racemate, provides deeper insight into why the double salt forms and how its behavior differs from that of the racemate. Besides, they support the expansion of solid‐phase candidates for new Mf APIs, thereby optimizing their pharmaceutical properties and overall performance.

## 2 Methodology

### 2.1 Materials

Mefloquine hydrochloride (purity > 98.0%), (S)‐Ketoprofen ((S)‐Kt), R/S‐Ketoprofen (Kt), (S)‐Ibuprofen ((S)‐Ib), and R/S‐Ibuprofen (Ib) were purchased from TCI Chem, Brazil. All chemicals were used as received without further purification.

### 2.2 Salt Preparation

The Mf salts with (S)‐Kt, Kt, (S)‐Ib and Ib were achieved through a direct acid–base reaction. First, the Mf free base was obtained from its hydrochloride salt as previously reported [27]. Then, all Mf salts were prepared using the same protocol: 50.0 mg (0.132 mmol) of the Mf free base was dissolved in 5 mL of an ethanol/water (3:1, v/v) mixture. Subsequently, 0.132 mmol of the acidic API was added, and the resulting mixture was stirred at 50°C for 20 min. The salts were obtained in high yield (approximately 90%). High‐quality crystals were grown by slowly evaporating the solvent over 4–5 days at room temperature.

### 2.3 Crystal Structure Determination

Single‐crystal X‐ray diffraction data for Mf‐S‐Kt and Mf‐S‐Ib were collected at the Manacá beamline of Sirius (Brazilian Synchrotron Light Laboratory, LNLS, Brazil) using a PILATUS2M detector and monochromatic radiation (λ = 0.670185 Å). Data for Mf‐Kt and Mf‐Ib were collected at room temperature on a Rigaku XtaLAB Synergy diffractometer equipped with a HyPix detector and a Cu microfocus source (λ = 1.54187 Å). Data reduction and multi‐scan absorption correction were carried out using CrysAlisPro software. All crystal structures were solved by SHELXT [28] and refined by full‐matrix least squares on *F* ^2^ using SHELXL [27] implemented in the Olex2 package [28]. Non‐hydrogen atoms were refined anisotropically, while hydrogen atoms bonded to N‐ and O‐atoms were located in the difference Fourier maps and refined as riding with isotropic displacement parameters set to 1.2 U_eq_(C) and 1.5 U_eq_(N/O).

### 2.4 Powder X‐Ray Diffraction (PXRD) Analysis

PXRD patterns were collected at room temperature using a Malvern Panalytical Empyrean diffractometer (Malvern, UK), equipped with a CuKα X‐ray tube (λ = 1.5418 Å) and a hybrid PIXcel3D detector. Data were acquired over a 2θ range of 5°–60° with a step size of 0.01° and a dwell time of 60 s per step. Automated variable divergence and anti‐scatter optics were used to precisely condition the beam on both the incident and diffracted X‐ray paths.

### 2.5 Thermal Analysis

Differential scanning calorimetry (DSC) and thermogravimetric analysis (TGA) were simultaneously performed using a NETZSCH STA 449F3 instrument. Approximately 20 mg of each sample was placed in a pinhole aluminum crucible and heated from 30°C to 350°C at a constant heating rate of 10°C⋅min^−1^ under a nitrogen atmosphere.

### 2.6 Solubility Experiments

The solubility of the Mf salts was determined in pure water using the flask saturation method. An excess amount of each salt was added to 5 mL of water, and the mixture was stirred at 22°C with a rotary mixer for 72 h to ensure equilibrium. After allowing the suspensions to settle for 2 h, the supernatant was filtered through a 0.22 μm syringe filter. The concentration of Mf in the filtrate was then quantified by HPLC analysis.

## 3 Results and Discussion

The propensity of Mf to form double salts with chiral organic acids [26], rather than the target diastereomeric salts, underscores a fundamental challenge in identifying an effective resolving agent. This prompted a shift toward a broader supramolecular landscape, specifically exploring the viability of drug–drug systems. Utilizing the NSAIDs Ketoprofen and Ibuprofen as chiral co‐formers is strategically advantageous due to their commercial availability in both racemic and enantiopure forms; however, the potential of these systems to yield stable diastereomeric phases remains uncharacterized. Beyond chiral resolution, these combinations offer a pathway to investigate potential synergistic effects between the two drugs that may enhance Mf's pharmacokinetic profile.

From a crystal engineering perspective, *the higher* basicity of Mf (pK_*a*_ = 8.6) compared to the acidity of Ib (pK_*a*_ = 4.4) and Kt (pK_*a*_ = 4.5) creates a sufficient ∆ pK_*a*_ to enable proton transfer. This ionization facilitates the formation of ionic salts via the deprotonation of the NSAID carboxyl group and the concomitant protonation of the Mf piperidine moiety. Furthermore, the pharmaceutical relevance of these co‐formers supports future scalability and regulatory alignment. Reacting racemic Mf with the (S)‐enantiomers of Kt and Ib has yielded double salts characterized by a lack of chiral discrimination, wherein the enantiopure coformer is incorporated into a lattice containing both Mf enantiomers. To establish a comparison, the pure racemate, derived from the reaction of racemic Mf with racemic NSAID counterparts, has also been isolated and characterized. A detailed description of the Mf double salts and their corresponding racemate structures is depicted below. Table 1 presents the crystallographic data for the structures.

**TABLE 1:** Crystallographic and structural refinement parameters of Mf–Ibuprofen and Mf–Ketoprofen systems.

Identification code	Mf‐S‐Kt	MF‐Kt	Mf‐S‐Ib	Mf‐Ib
Empirical formula	C_66_H_64_F_12_N_4_O_10_	C_33_H_32_ F_6_ N_2_O_5_	C_30_H_33_F_6_N_2_O_3_	C_30_H_34_F_6_N_2_O_3_
Formula weight	650.60	650.60	584.59	584.59
Temperature/K	100.0	298.0(2)	100.0	298.0
Crystal system	Monoclinic	Monoclinic	Monoclinic	Monoclinic
Space group	*P*2	*P*2/*n*	*P*2_1_	*P*2_1_/*c*
*a*/Å	19.920(6)	20.1836(6)	12.940(3)	13.5649(6)
*b*/Å	7.910(5)	7.8621(2)	7.200(3)	7.0881(3)
*c*/Å	20.230(9)	20.6068(6)	31.130(3)	31.2841(11)
*β*/°	103.07(2)	101.410(3)	91.480(9)	92.816(4)
Volume/Å^3^	3105(3)	3205.37(16)	2899.4(13)	3004.3(2)
*Z*	2	4	4	4
*ρ*_*calc*_ g/cm^3^	1.392	1.348	1.339	1.292
*μ*/mm^−1^	0.103	0.979	0.098	0.927
*F*(000)	1352.0	1352.0	1224.0	1224.0
Crystal size/mm^3^	0.05 × 0.041 × 0.016	0.261 × 0.15617 × 0.12	0.06 × 0.04 × 0.038	0.072 × 0.057 × 0.042
Radiation	Synchrotron (*λ* = 0.670185)	CuK*α* (*λ* = 1.54184)	Synchrotron (*λ* = 0.67019)	CuK*α* (*λ* = 1.54184)
2θ range for data collection/°	1.98 to 60.178	5.6 to 160.202	3.91 to 51.592	5.656 to 160.458
Index ranges	−28 ≤ h ≤ 28, −11 ≤ k ≤ 11, −29 ≤ l ≤ 28	−25 ≤ h ≤ 25, −9 ≤ k ≤ 6, −26 ≤ l ≤ 25	−16 ≤ h ≤ 16, −9 ≤ k ≤ 9, −40 ≤ l ≤ 40	−15 ≤ h ≤ 17, −8 ≤ k ≤ 8, −37 ≤ l ≤ 39
Reflections collected	68,493	34,813	84,531	23,668
Independent reflections	18,061 [*R*_int_ = 0.0312, *R*_sigma_ = 0.0264]	6868 [*R*_int_ = 0.0359, *R*_sigma_ = 0.0282]	13,283 [*R*_int_ = 0.0367, *R*_sigma_ = 0.0250]	6183 [*R*_int_ = 0.0320, *R*_sigma_ = 0.0270]
Data/restraints/parameters	18,061/1/839	6868/0/417	13,283/1/747	6183/0/406
Goodness‐of‐fit on *F* ^2^	1.061	1.073	1.074	1.046
Final R indexes [*I* > = 2*σ* (*I*)]	*R*_1_ = 0.0351, *wR*_2_ = 0.0946	*R*_1_ = 0.0692, *wR*_2_ = 0.2100	*R*_1_ = 0.0357, *wR*_2_ = 0.1012	*R*_1_ = 0.0588, *wR*_2_ = 0.1710
Final *R* indexes [all data]	*R*_1_ = 0.0355, *wR*_2_ = 0.0952	*R*_1_ = 0.0837, *wR*_2_ = 0.2277	*R*_1_ = 0.0366, *wR*_2_ = 0.1021	*R*_1_ = 0.0857, *wR*_2_ = 0.1934
Largest diff. Peak/hole/*e* Å^−3^	0.52/−0.55	0.64/−0.30	0.34/−0.24	0.37/−0.20
Flack parameter	−0.02(7)	—	0.04(9)	—
CCDC N.	2,481,598	2,481,512	2,481,619	2,481,519

### 3.1 Mf‐(S)‐Ketoprofen (Mf‐S‐Kt)

**FIGURE 1:**
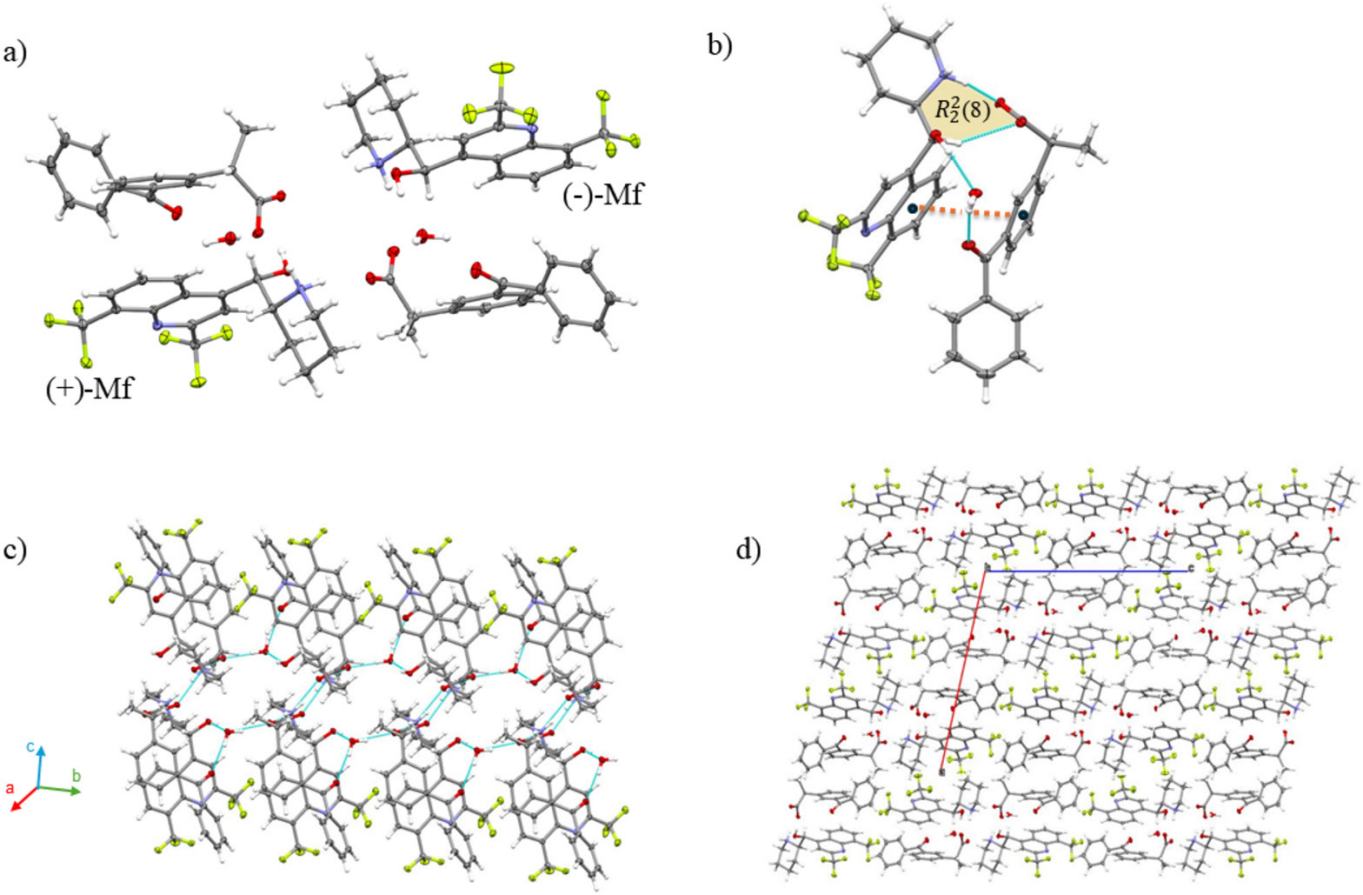
(a) The asu content of Mf‐S‐Kt. (b) The cation–anion assembly of the ionic pair containing the (−)‐Mf enantiomer. The assembly is stabilized by the $R_{2}^{2}$(8) synthon (highlighted), featuring NH···O (2.697(3),167.0(1)°) H‐bonds and CH···O (3.289(2)Å, 139.0(1)°) interactions, and further by π···π interactions (dashed orange line; 4.038(2) Å, 16.70(3)). An analogous arrangement is observed in the structure for the (+)‐Mf enantiomer. (c) Bilayer structural motif formed through the assembly of these ion pairs. (d) Crystal packing of Mf‐S‐Kt.

The Mf‐S‐Kt double salt crystallizes as prismatic crystals (See ESI) within the Sohncke space group *P*2 with *Z*' = 2 (Table 1). The asymmetric unit (asu) contains both (+)‐ and (−)‐Mf^+^ cations, two enantiopure (S)‐Kt^−^ anions, and two water molecules (Figure 1a).

In the Mf‐S‐Kt structure, the conformations of the Mf^+^ cations are equivalent by inversion symmetry, while the anions adopt distinct conformations (Figure S1). This variation may be a response to adapting and recognizing cations with different chiral handedness. The cations interact with the anions through an NH^+^•••COO^−^ H‐bond, forming an ionic pair where the molecules are aligned side by side and stabilized by π•••π interactions involving their aromatic rings (Figure S2). Each independent ionic pair self‐assembles into a 1D chain, bridged by a water molecule (Figure 1b). The spatial orientation of these constituent molecules results in the formation of homochiral cationic and anionic layers along the chain motif. Furthermore, the 1D ionic chains comprising cations of opposite handedness are integrated into a bilayer architecture, stabilized by inter‐chain NH•••O H‐bonds between the ammonium and carboxyl moieties of adjacent ion pairs. In this structural motif, the chains are oriented relative to each other such that, if the anion were not homochiral and conformationally distinct, they would be related by an inversion center. This bilayer extends along the [010] direction, with the hydrophobic moiety of both cations and anions facing outward. The bilayers are then packed into a 3D zigzag network via CH•••F interactions, yielding the packing arrangement shown in Figure 1d.

### 3.2 Mf‐Ketoprofen (Mf‐Kt)

**FIGURE 2:**
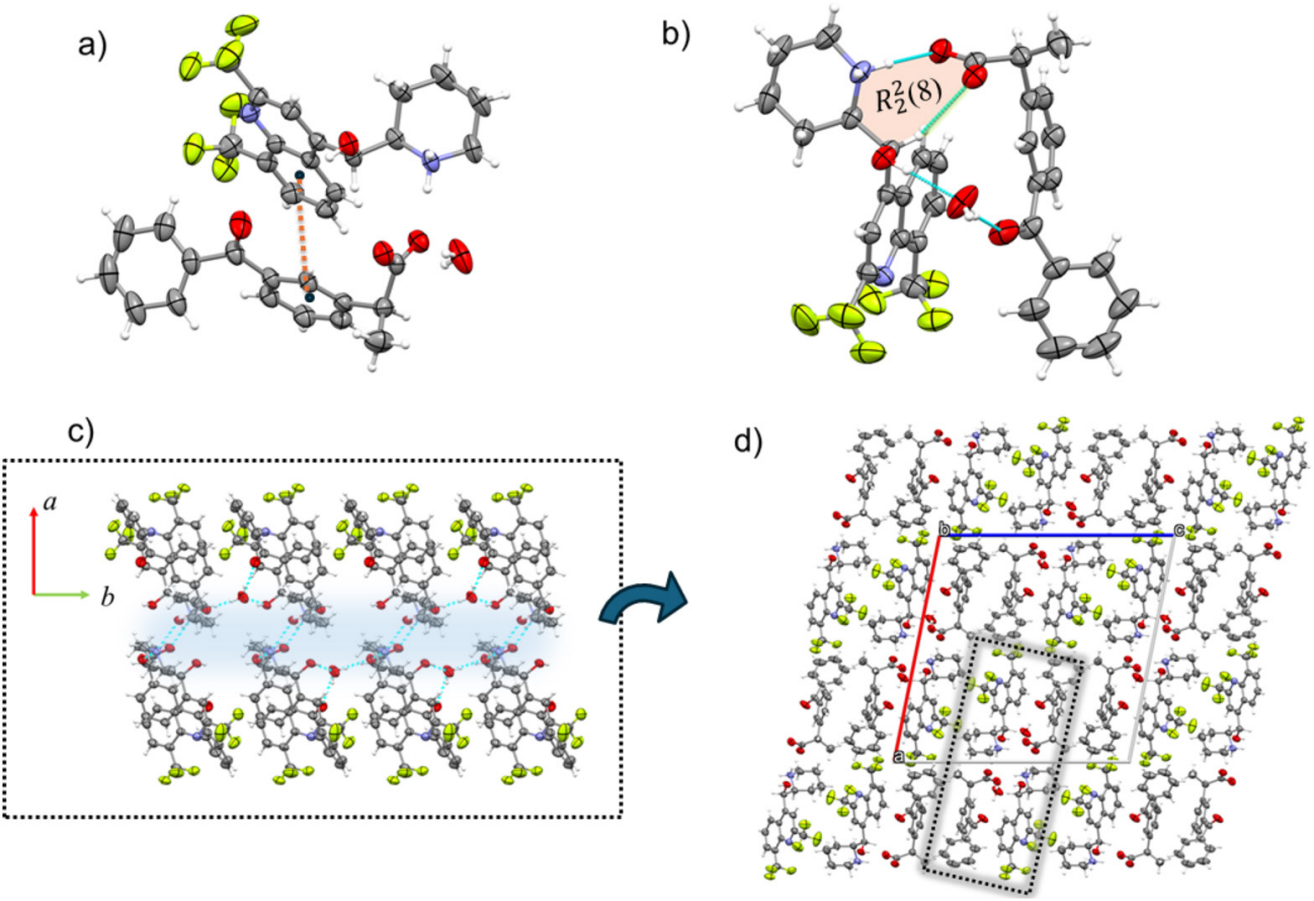
(a) Asu of Mf‐Kt. (b) The cation‐anion assembly highlighting the $R_{2}^{2}$(8) synthon formed via NH···O (2.727(3) Å, 159.3(2)) H‐bonds and CH···O (3.391(3) Å, 144.8(2)°) interaction. Furthermore, the assembly is stabilized by π···π interactions (dashed orange line; 4.075 Å, 18.16°). Similar motifs were also observed in Mf‐S‐Kt. (c) Bilayer structural motif formed by the centrosymmetric association of ionic pairs. The structure features a hydrophobic exterior of nonpolar groups protecting a central hydrophilic polar domain (in blue) (d) Crystal packing showing the assembly of these motifs (highlighted in gray).

The racemic Mf‐Kt salt forms prism‐like crystals within the centrosymmetric space group P2/n (Table 1). Mf‐Kt is isomorphous and isostructural with Mf‐S‐Kt, while having *Z*' = 1. The asu consists of a single mefloquinium cation, a ketoprofen anion, and one water molecule, as illustrated in Figure 2a.

In the crystal, the Mf^+^ cation binds to the Kt^−^ anion through an NH^+^···COO^−^ H‐bond, arranging the aromatic groups of cations and anions on the same side and further stabilized by π···π interactions (Figure 2b). This arrangement closely resembles the one observed in the Mf‐S‐Kt structure. The ionic pairs assemble into discrete dimers through the NH•••COO^−^ H‐bonds, characterized by centrosymmetric $R_{4}^{4}$(12) synthon notation. These dimers are further interconnected by OH•••O hydrogen bonds involving water molecules, leading to the formation of a double chain that extends along the [010] direction. Along this chain, adjacent ionic pairs of opposite handedness are related via inversion centers. In the Mf‐S‐Kt counterpart salt, this structural motif gives rise to homochiral cationic chains throughout the structure. Through bilayer motifs, nonpolar groups are oriented outward, enclosing a central polar region (Figure 2c). Along the [1¯01] direction, these motifs assemble each other through CH•••F interactions, resulting in a packing arrangement similar to that observed in Mf‐S‐Kt (Figure 2d). Consequently, in the *bc*‐plane, the bilayers adopt a zigzag configuration extending in the [001] direction. As a result, the Mf‐Kt structure exhibits a spatial distribution of polar domains throughout the structure.

### 3.3 Mf‐(S)‐Ibuprofen (Mf‐S‐Ib)

**FIGURE 3:**
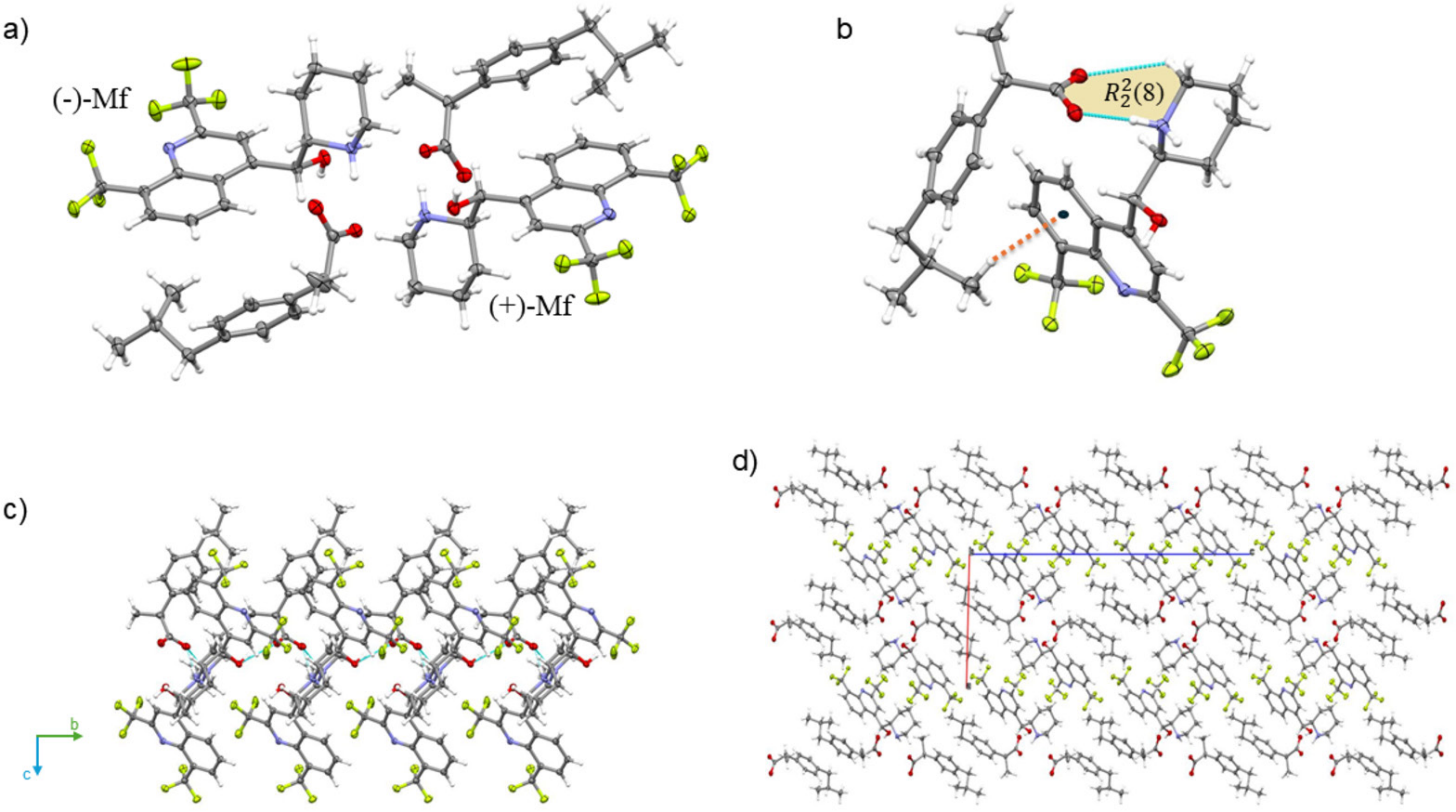
(a) The content asu of Mf‐S‐Ib. (b) The cation‐anion assembly highlighting the $R_{2}^{2}$(8) synthon formed via NH···O (2.692(2) Å, 175.0(1)°) H‐bonds and CH···O (3.295(3) Å, 120.5(1)°) interaction. Furthermore, the assembly is stabilized by CH···π interactions (dashed orange line; 3.641(2) Å, 122.1(2)°). (c) Bilayer structural motif formed by the association of ionic pairs. (d) Crystal packing showing the assembly of these motifs (highlighted in gray).

The Mf‐Ib crystallizes as prism‐like crystals (See ESI) in the Sohncke space group *P*2_1_, with *Z′* = 2 (Table 1). The asu comprises two protonated Mf with opposite handedness and two (S)‐Ib^−^ anions (Figure 3a).

In the structure, both Mf^+^ enantiomers exhibit equivalent conformations (due to inversion symmetry) while the anions adopt the same conformation (Figure S3). Cations and anions form face‐to‐face ion pairs through NH···COO^−^ H‐bonds. Within these pairs, the mean planes of the respective aromatic rings are mutually inclined at a dihedral angle of 53.89(2)°. This arrangement is further stabilized by C–H⋅⋅⋅π interactions involving the terminal methyl groups and the aromatic moiety of the Mf^+^ cation (Figure 3c). These interactions occur both between the terminal part of the anion and the aromatic section of the cation, as well as between the central part of the cation and the aromatic ring of the anion. Unlike the double salt Mf‐S‐Kt, the independent ionic pairs in Mf‐S‐Ib are assembled via an $R_{4}^{2}$(8) H‐bonding synthon to form cyclic units. These units are further connected through OH•••COO^−^ H‐bonds, creating a 1D chain. This arrangement forms a compact, hydrophilic region surrounded by cations. Along the chain, the Mf enantiomers are aligned on the same side, resulting in homochiral chains throughout the crystal. The overall packing of Mf‐S‐Ib is achieved through the assembly of these chains. Along the [100] direction, adjacent chains are offset relative to each other and stabilized by CH•••F interactions.

### 3.4 Mf‐Ibuprofen (Mf‐Ib)

**FIGURE 4:**
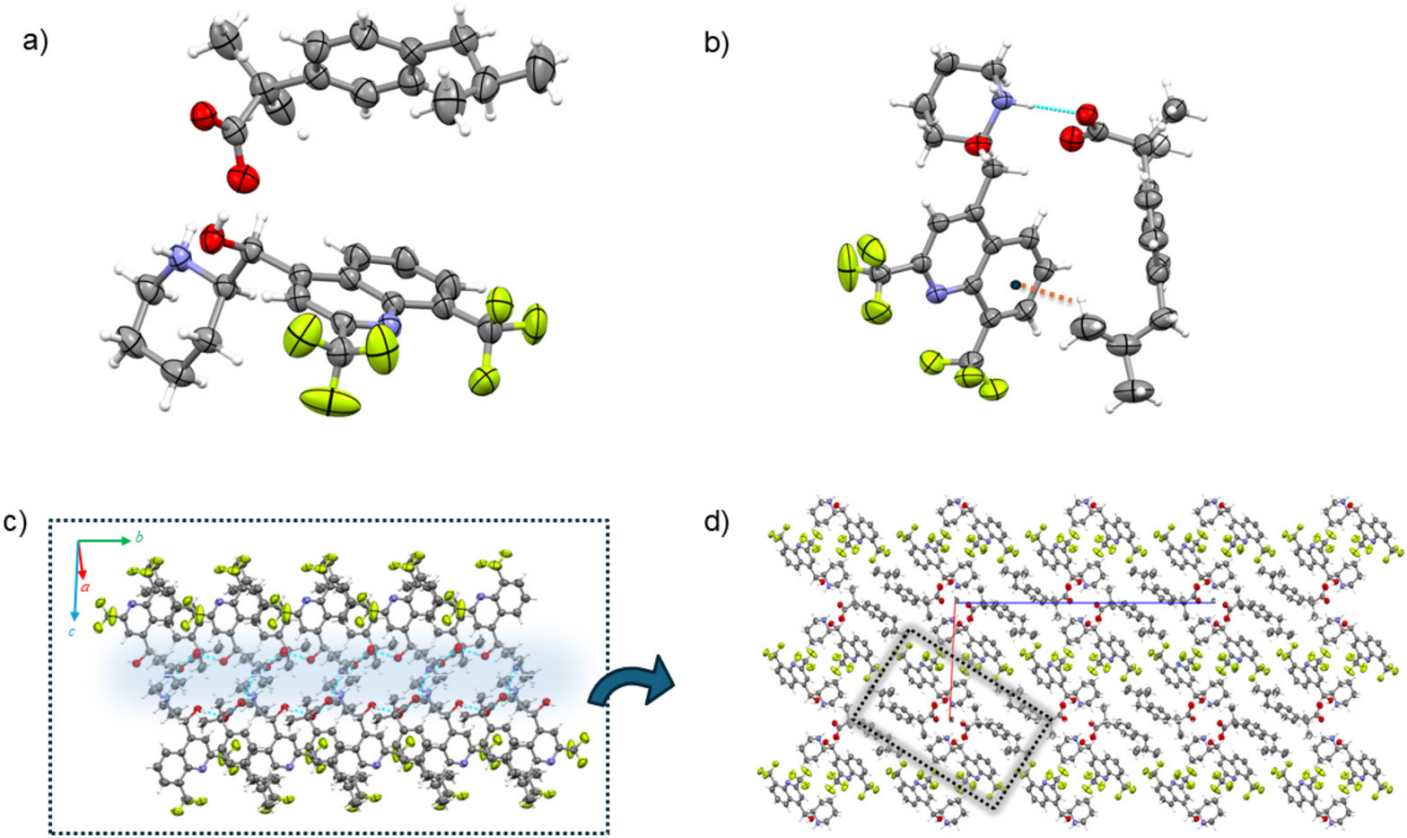
The crystal structure of Mf‐Ib. (a) Representative contents of the asu. (b) Unlike Mf‐S‐Ib, the cation–anion framework is stabilized by a single NH⋅⋅⋅O H‐bond (2.7711(3) Å, 177.9(1)°). (c) The ionic pairs assemble centrosymmetrically to form a bilayer chain, with the polar regions (shown in blue) facing inward and the hydrophobic regions directed outward. (d) The Mf‐Ib packing is characterized by the stacking of these bilayers (in gray).

The reaction between Mf and racemic Ib yields crystals that belong to the monoclinic space group *P*2_*1*_ */c*. The asu comprises one mefloquinium cation and one Ib^−^ anion, which are linked via an NH···COO^−^ hydrogen bond (Figure 4a).

The Ib^−^ anion is disordered over two opposite enantiomeric configurations, with an occupancy ratio of 20:80. Due to the crystal's centrosymmetric structure and the presence of enantiomeric disorder, the compound is classified as a racemic solid solution. Unlike the ketoprofen systems, the Mf‐S‐Ib and Mf‐Ib salts are not isomorphic. In Mf‐Ib, the cation and anion associate into an ionic pair via NH···COO^−^ H‐bonds (Figure 4b). These pairs further interact through centrosymmetric OH···COO^−^ H‐bonds, leading to dimer formation (Figure S4). The dimers align along the [010] direction, creating a double‐chain motif (Figure 4c). The overall packing consists of stacked double layers that are connected in the ac plane by CH···F interactions between adjacent cations and CH···π interactions involving the tert‐butyl groups and the aromatic rings of the anions. This arrangement results in a compact and stabilized three‐dimensional structure, as illustrated in Figure 4d.

### 3.5 Phase Relation

Comparing a double salt to its corresponding racemate provides a valuable method for studying enantiomer discrimination and its underlying structural basis. In the context of diastereoisomeric resolution via crystallization, the formation of a double salt indicates a failure of chiral recognition. It led to a non‐centrosymmetric crystal lattice in which both enantiomers of the target compound cocrystallize with an enantiopure coformer (Figure S5). This arrangement imposes a distinct supramolecular constraint: both Mf enantiomers are incorporated into the chiral environment created by the (S)‐coformer, avoiding any enantioselective preference. The resulting structural complexity and lack of symmetry lead to *Z*' > 1 structures.

The Mf‐Kt racemate is isostructural with the Mf‐(S)‐Kt double salt but crystallizes with *Z*' = 1. As expected, given the presence of inversion symmetry between its components, which is not fulfilled in the double salt structures, the *Z*' value for the racemate is lower than that of the Mf‐S‐Kt double salt. Additionally, it shows that both Mf enantiomers interact with (S)‐ketoprofen through analogous interactions. This observation has also been observed in other Mf double salts [26], which suggests a broader trend in which double salts adopt a chiral packing arrangement that closely mimics the centrosymmetric packing of their corresponding racemates. An overlay of the packing of these phases illustrates this similarity (see Figure S6).

However, Mf‐S‐Ib and Mf‐Ib have similar unit cells but differ in their architecture. Mf‐Ib is a solid solution of the coformer, indicating that cation‐anion recognition is so similar that they form a single solid‐state phase. Unlike ketoprofen salts, there is no perfect match between the packing of the Ib and (S)‐Ib phases with Mf.

The double salts display homochiral domains that assemble into a complete racemic structure. This feature maintains significant isostructurality with the corresponding racemic crystal system. Therefore, selecting a suitable resolving agent for Mf involves enantiomeric discrimination, which is accomplished by creating homochiral domains that are structurally uncorrelated with the racemic phase.

**FIGURE 5:**
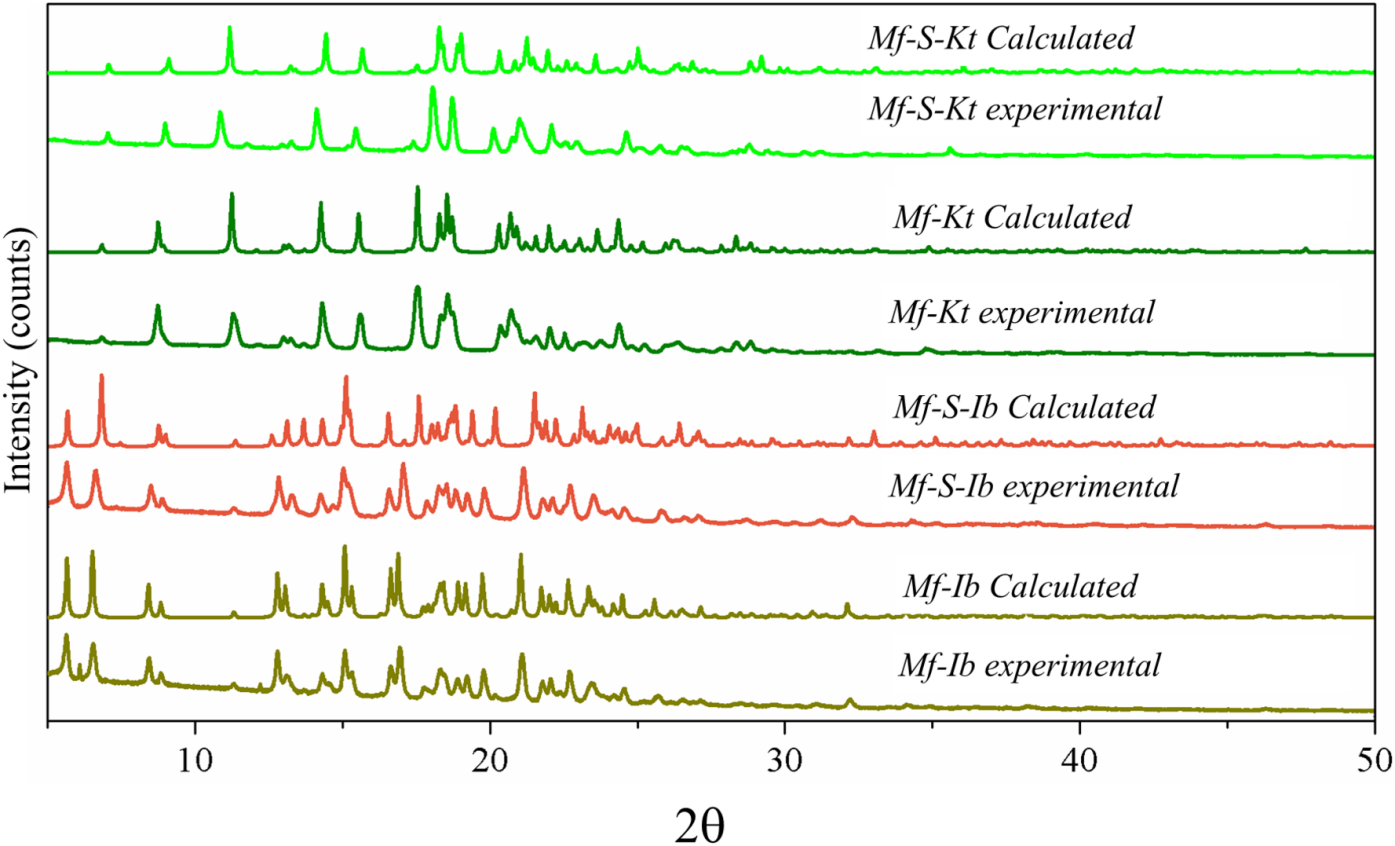
Experimental and calculated diffractograms of Mf phases.

PXRD analysis has been used to assess the structural similarities and authenticity of both the double salt and racemate phases. Figure 5 shows experimental and calculated patterns derived from the scxrd data.

All experimental diffractograms match their corresponding calculated patterns, confirming that they were produced with high purity and phase authenticity. The phase homogeneity for the Mf‐(S)‐Kt and Mf‐(S)‐Ib indicates that double salt formation occurred throughout the entire system. Comparing the patterns of double salts and racemates shows that, although they align in specific angular ranges (between 5° and 17.66° for the ketoprofen salts and between 5° and 12° for the ibuprofen salts), there are noticeable differences in other regions. This partial overlap supports the idea that the double salt structure resembles that of the racemate, suggesting they share structural motifs.

**FIGURE 6:**
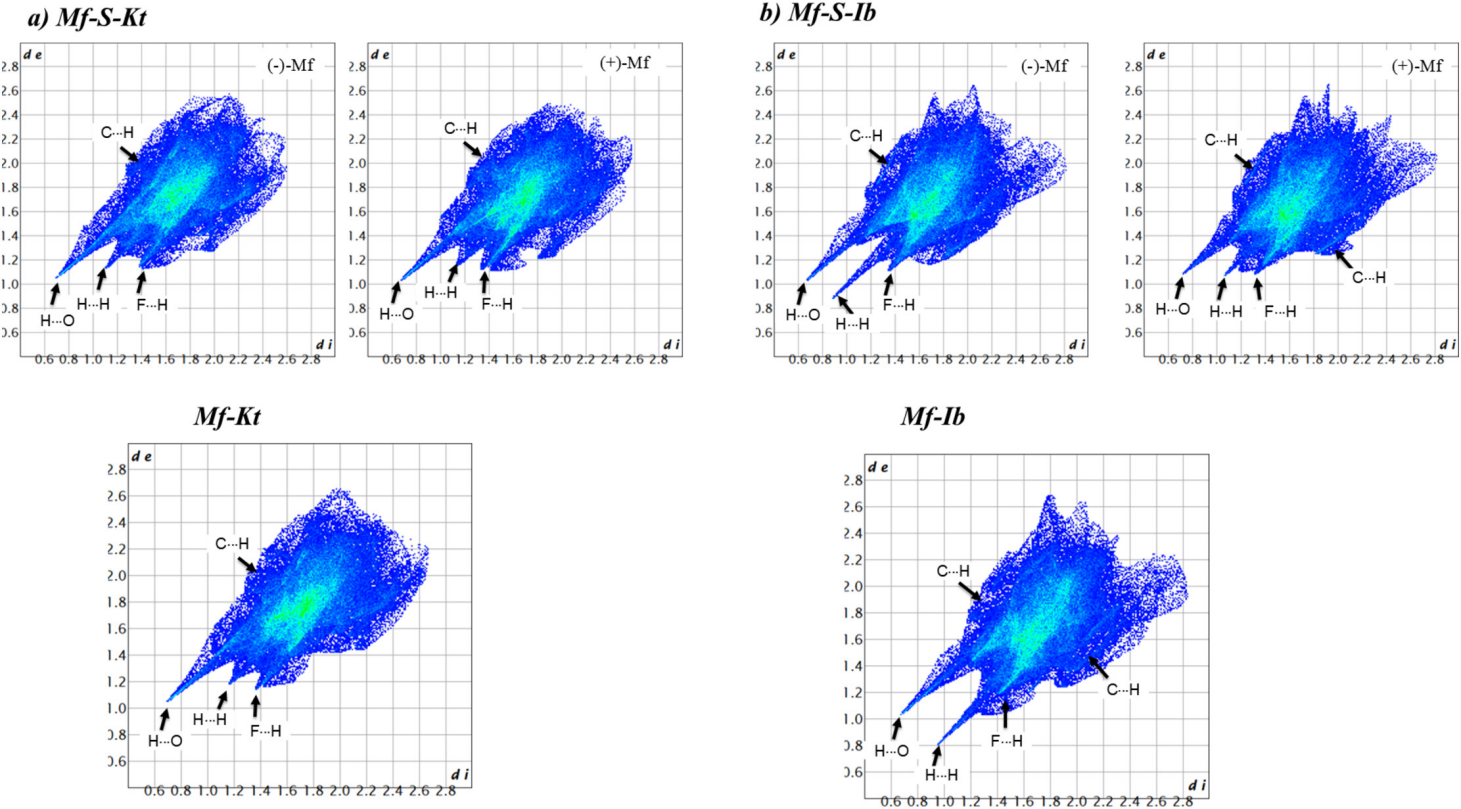
2D‐fingerprint plots for (a) Mf‐S‐Kt and Mf‐Kt, and (b) Mf‐S‐Ib and Mf‐Ib.

Hirshfeld surface (HS) analysis and 2D fingerprint plots (Figure 6) have been further used to examine the structural similarities among the compounds.

The 2D plots of the Mf^+^ enantiomers in the Mf‐S‐Kt salt are nearly identical, indicating that both enantiomers interact with the (S)‐Kt cation through similar intermolecular forces. Consistent with their isostructural nature, the Mf^+^ cations in both the racemate and the double salt have comparable supramolecular environments. This similarity is evident in their 2D plots, which closely resemble each other in shape and (*d*_i_, *d*_*e*_) distribution. The graphs reveal sharp peaks at *d*_i_ + *d*_*e*_ ~ 2.6 Å, indicating H···O contacts from NH···O and O_*w*_H···O H‐bonds. These account for 12.5% and 11.7% of the HS in the (−)‐Mf and (+)‐Mf cations of the double salt, respectively, and 11,6% in the racemate. H···H contacts contribute 26.7% and 27.2% for (−)‐Mf and (+)‐Mf (double salt), respectively, and 28,6% for the racemate. In contrast, the 2D plots for the Mf‐Ib and Mf‐S‐Ib salts show minor differences. In the Mf‐S‐Ib double salts, the primary distinction between the Mf enantiomers is a close H···H contact. The (−)‐Mf enantiomer exhibits a higher percentage of O···H contacts (10.8% versus 9.5%) and a slightly lower percentage of H···H contacts (25.4% versus 26.8%) compared to the (−)‐Mf enantiomer (Figure S7). Despite these slight variations, the overall molecular packing arrangement for both enantiomers is very similar and primarily driven by H···H contacts. Interestingly, the 2D fingerprint of the racemate shows a similarity to that of one of the enantiomers in the double salt. This observation suggests that the miscibility of the ion pairs within the solid solution results in an interaction environment that closely resembles that experienced by this specific enantiomer.

The thermal analysis also highlights the structural similarities between the phases.

**FIGURE 7:**
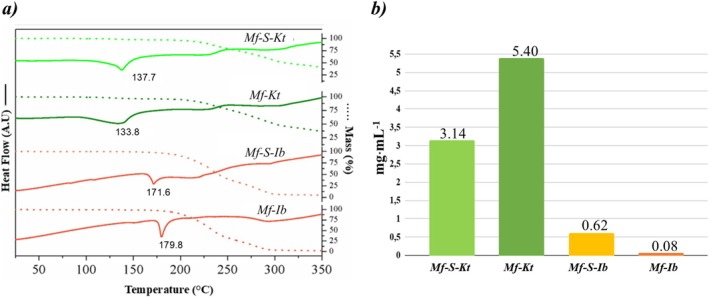
(a) DSC/TGA curves of solubility and (b) water solubility of Mf salts.

Figure 7 a displays the DSC/TGA curves that demonstrate these thermal behaviors for both the racemic crystal pairs and the double salts.

Comparing the DSC/TGA curves of the Mf‐S‐Kt and Mf‐S‐Ib double salts with those of their corresponding racemates indicates that the double salts are as thermally stable as their racemates. For the Kt salts, the similarity in thermal profiles is attributed to their isostructural nature; the double salt and the racemate exist in analogous supramolecular environments, leading to nearly identical decomposition and phase‐transition pathways. Both Mf‐S‐Kt and Mf‐Kt undergo dehydration within a similar temperature range before degradation begins. The DSC curves for both species exhibit a discrete endothermic event at 137.3°C, corresponding to water loss, which is corroborated by a slight mass loss in the TGA curve. Above 200°C, the TGA curves show a continuous mass loss, indicating the onset of thermodegradation. This process is reflected in the DSC thermograms as broad, ill‐defined exothermic fluctuations, characteristic of complex decomposition.

Although not isomorphic, Mf‐S‐Ib and Mf‐Ib show isostructural features and similar thermal behavior, as observed with previously described Mefloquine‐Ketoprofen systems. Upon heating, both samples undergo an initial dehydration phase, followed by thermal decomposition. The DSC curve shows an endothermic peak at 171.9°C for Mf‐S‐Ib and 179.6°C for Mf‐Ib. These events are associated with mass loss on the TGA curves, confirming the loss of water or solvent molecules. After dehydration, thermal degradation begins at approximately 185°C, as evidenced by distinct profiles in the DSC/TGA curves for both compounds.

Thermodynamically, solubility reflects the balance between the lattice energy and the solvation energy released during particle‐solvent interactions. Consequently, solubility serves as a reliable indicator of the relative thermodynamic stability of Mf salts. Experimental data show that Mf‐Kt has 1.7 times the solubility of Mf‐S‐Kt, suggesting that the double salt is more stable than the corresponding racemate. It indicates that including a complementary anion enantiomer within the racemate lattice effectively enhances the racemate's solubility. This contrasts with the ibuprofen salts, where Mf‐Ib is 8.1 times less soluble than Mf‐S‐Ib, i.e., the racemate is more stable than the double salt, highlighting how differences in molecular packing influence the relative solubility trends.

Although the drug–drug approach with NDAIS has not led to enantiomer discrimination by forming diastereomeric Mf phases, the solid forms reported in this study nonetheless expand the scope of Mf solid‐state chemistry, adding to the potential pharmaceutical solid forms. The Mf‐S‐Kt demonstrates 1.24 times the solubility of the clinically used (±)‐ Mf HCl salt. This increase in solubility may be relevant for improving drug bioavailability. This phase forms a drug–drug system that may provide synergistic benefits by combining Mf and (S)‐Kt. Its properties make it a candidate for drug development, but thorough pharmacokinetic and pharmacodynamic studies are necessary to confirm its safety, efficacy, and potential advantages over current treatments.

## 4 Conclusions

This study highlights the crystallization behavior of racemic Mf with (S)‐Kt, Kt, (S)‐Ib, and Ib within a drug–drug approach. The results show that double salts often mimic the supramolecular organization of their corresponding racemates, underscoring the lack of enantiomeric recognition in these systems. In the ketoprofen systems, both the racemate and the double salt were isostructural and isomorphic. In contrast, in the Mf‐Ib system, distinct packing arrangements emerged despite similar unit cells, with the racemic phase exhibiting solid‐solution characteristics. Structural analyses revealed that structural mimicry translates into comparable physicochemical properties between racemates and double salts. Nonetheless, solubility trends diverged between the two systems: while the Mf‐Kt racemate exhibited higher solubility than its double salt, the inverse was observed for the Mf‐Ib system. Notably, the Mf‐S‐Kt phase showed greater solubility than the marketed hydrochloride salt, suggesting potential for improved bioavailability. Altogether, these findings reinforce the inherent unpredictability of enantiomeric resolution through salt formation with chiral coformers. At the same time, they expand the solid‐form landscape of mefloquine and highlight promising candidates, particularly the Mf‐S‐Kt salt, for future pharmaceutical exploration.

## Funding

This work was supported by the Fundação de Apoio ao Desenvolvimento do Ensino, Ciência e Tecnologia do Estado de Mato Grosso do Sul (#TO N. 123/2024) and the Laboratório Nacional de Luz Síncrotron (20233394).

## Supporting information


**Figure S1:** Superposition of the independent (S)‐Keto anion conformations in Mf‐S‐Kt was performed via the propionate moiety. It is noted that the phenyl group exhibits a rotational difference among the molecules.
**Figure S2:** Ionic pair arrangement of the independent Mf^+^ enantiomer with the S‐ketoprofen anion in the structure of Mf–S‐Kt.
**Figure S3:** Superposition of the crystallographically independent (S)‐Ib anions. (b) Overlay of the (+)‐Mf and inverted (−)‐Mf conformations, within the Mf‐S‐Ibu structure.
**Figure S4:** Dimeric arrangement of ionic pairs in the Mf‐Ib structure.
**Figure S5:** ADDSYM analyses in Platon for (a) Mf‐S‐Kt and (b) Mf‐S‐Ib. Although the *P*2/n and *P*2_1_/c space groups were proposed for Mf‐S‐Kt and Mf‐S‐Ib, respectively, these are incompatible with the presence of enantiopure anions within the crystal lattice. Such pseudo‐symmetry is a frequent challenge in the characterization of double salts. Attempts to refine these structures within centrosymmetric space groups led to significant anionic disorder and failed to achieve model convergence.
**Figure S6:** Overlay of the crystal packing arrangements of Mf‐S‐Kt and Mf‐Kt.
**Figure S7:** Percentage of intermolecular contact in the salts.

## Data Availability

The data that support the findings of this study are available on request from the corresponding author. The data are not publicly available due to privacy or ethical restrictions.
