# Supplementary material for: Double Salts and Racemate in Mefloquine–Ibuprofen and Mefloquine–Ketoprofen Systems. A Structural and Thermochemical Analysis
Source: Chirality. 2026 Feb 13;38(3):e70088. doi: 10.1002/chir.70088 (PMC12903187; doi:10.1002/chir.70088)
Supplement: Supplementary file 1 — Figure S1: Superposition of the independent (S)‐Keto anion conformations in Mf‐S‐Kt was performed via the propionate moiety. It is noted that the phenyl group exhibits a rotational difference among the molecules. Figure S2: Ionic pair arrangement of the independent Mf+ enantiomer with the S‐ketoprofen anion in the structure of Mf–S‐Kt. Figure S3: Superposition of the crystallographically independent (S)‐Ib anions. (b) Overlay of the (+)‐Mf and inverted (−)‐Mf conformations, within the Mf‐S‐Ibu structure. Figure S4: Dimeric arrangement of ionic pairs in the Mf‐Ib structure. Figure S5: ADDSYM analyses in Platon for (a) Mf‐S‐Kt and (b) Mf‐S‐Ib. Although the P2/n and P21/c space groups were proposed for Mf‐S‐Kt and Mf‐S‐Ib, respectively, these are incompatible with the presence of enantiopure anions within the crystal lattice. Such pseudo‐symmetry is a frequent challenge in the characterization of double salts. Attempts to refine these structures within centrosymmetric space groups led to significant anionic disorder and failed to achieve model convergence. Figure S6: Overlay of the crystal packing arrangements of Mf‐S‐Kt and Mf‐Kt. Figure S7: Percentage of intermolecular contact in the salts. [file CHIR-38-e70088-s001.docx]

**SUPPORTING INFORMATION**

**Double Salts and Racemate in Mefloquine–Ibuprofen and Mefloquine–Ketoprofen Systems. A Structural and thermochemical analysis**

*Juliana Martins^a^, João V. Segatto^a^, Juan C. Tenorio^b^, and Paulo S. Carvalho Jr^a^*

*^a^* Institute of Physics, Federal University of Mato Grosso do Sul, 79070-900, Campo Grande, MS, Brazil. *^*^e-mail: [paulo.sousa@ufms.br](mailto:paulo.sousa@ufms.br)

*^b^* Physics Institute, Universidade Federal do Rio de Janeiro, 21941-909, Rio de Janeiro, RJ, Brazil.

**COMPLEMENTARY FIGURES AND TABLES**

**Figure S1**. Superposition of the independent (S)-Keto anion conformations in Mf-S-Kt was performed via the propionate moiety. It is noted that the phenyl group exhibits a rotational difference among the molecules.


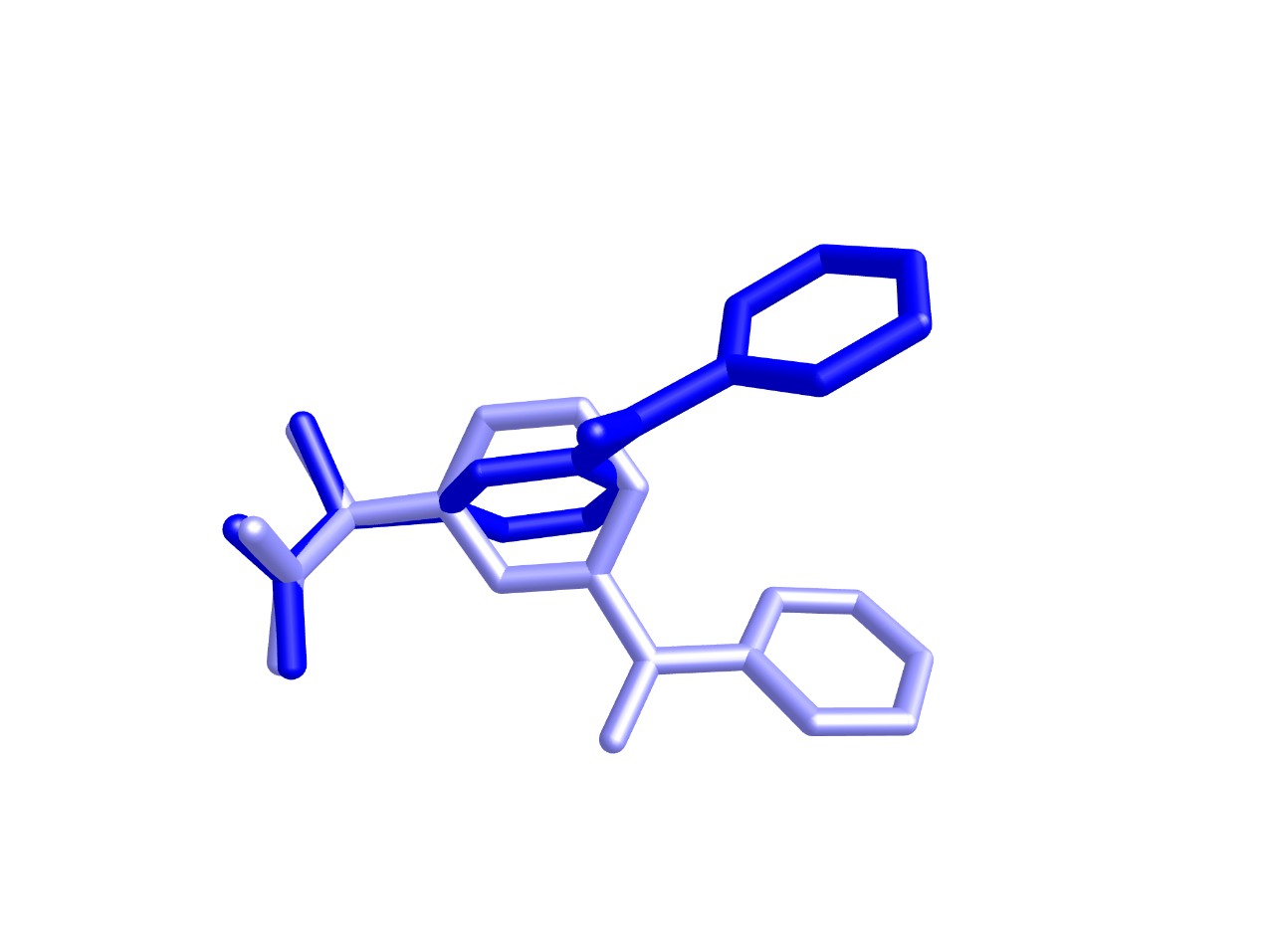


**Figure S2.** Ionic pair arrangement of the independent Mf⁺ enantiomer with the S-ketoprofen anion in the structure of Mf–S-Kt.


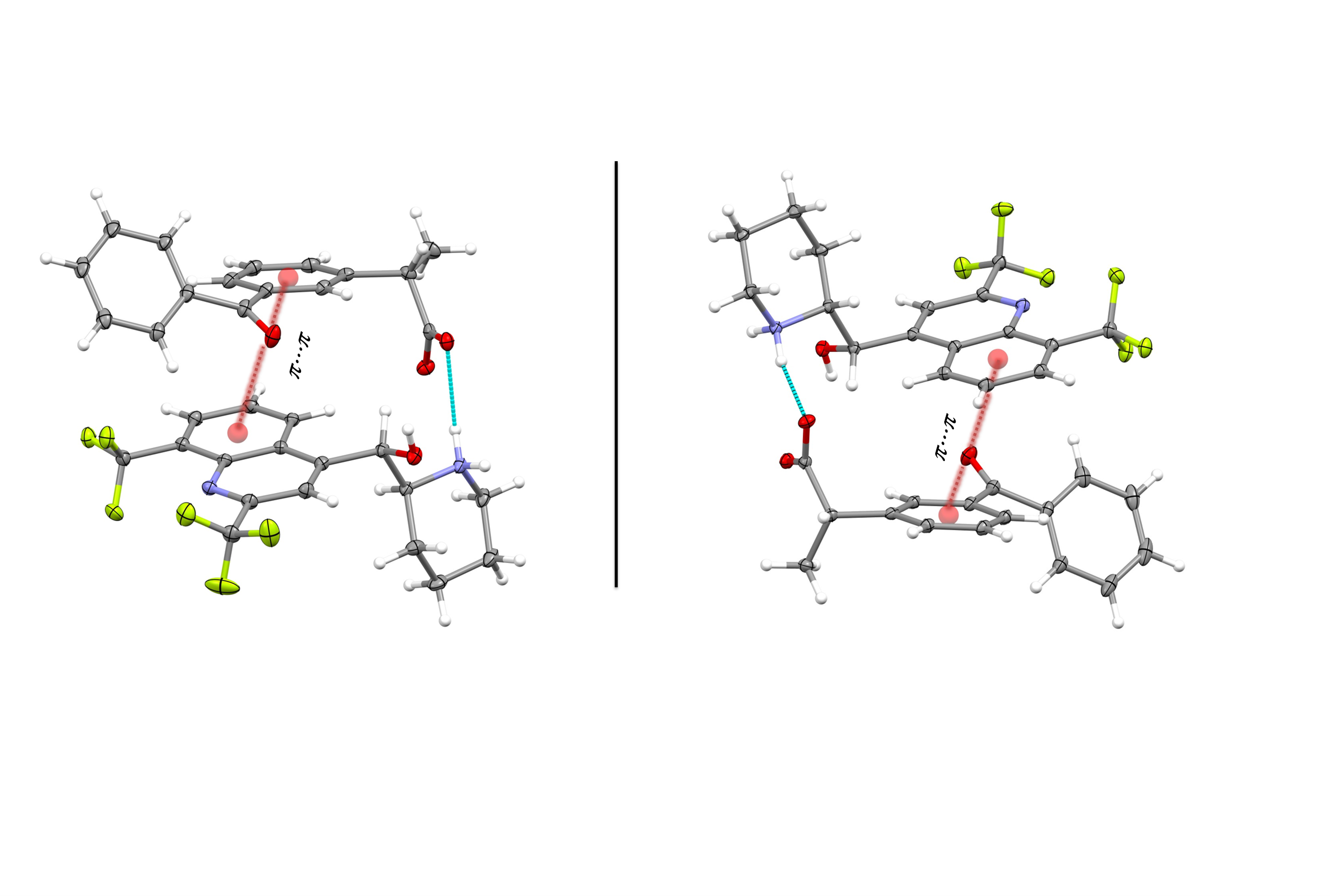


**Figure S3. Superposition** of the crystallographically independent (S)-Ib anions. **(b) Overlay** of the (+)-Mf and inverted (-)-Mf conformations, within the Mf-S-Ibu structure.

| 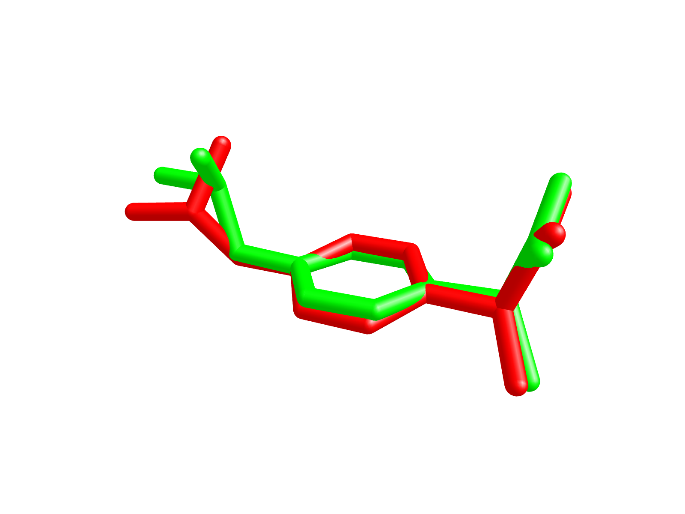 | 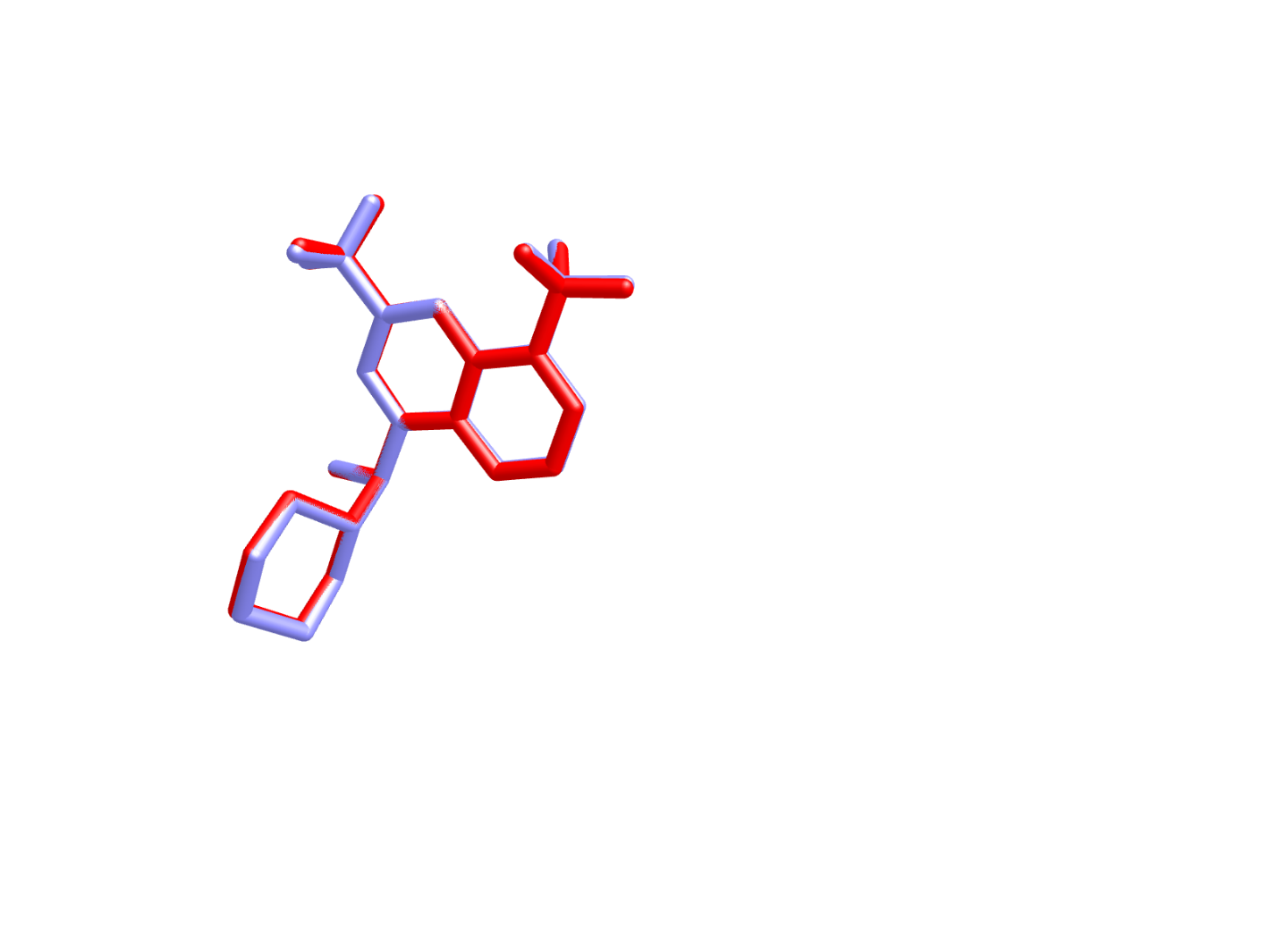 |
| --- | --- |
|  |  |

**Figure S4.** Dimeric arrangement of ionic pairs in the Mf-Ib structure.


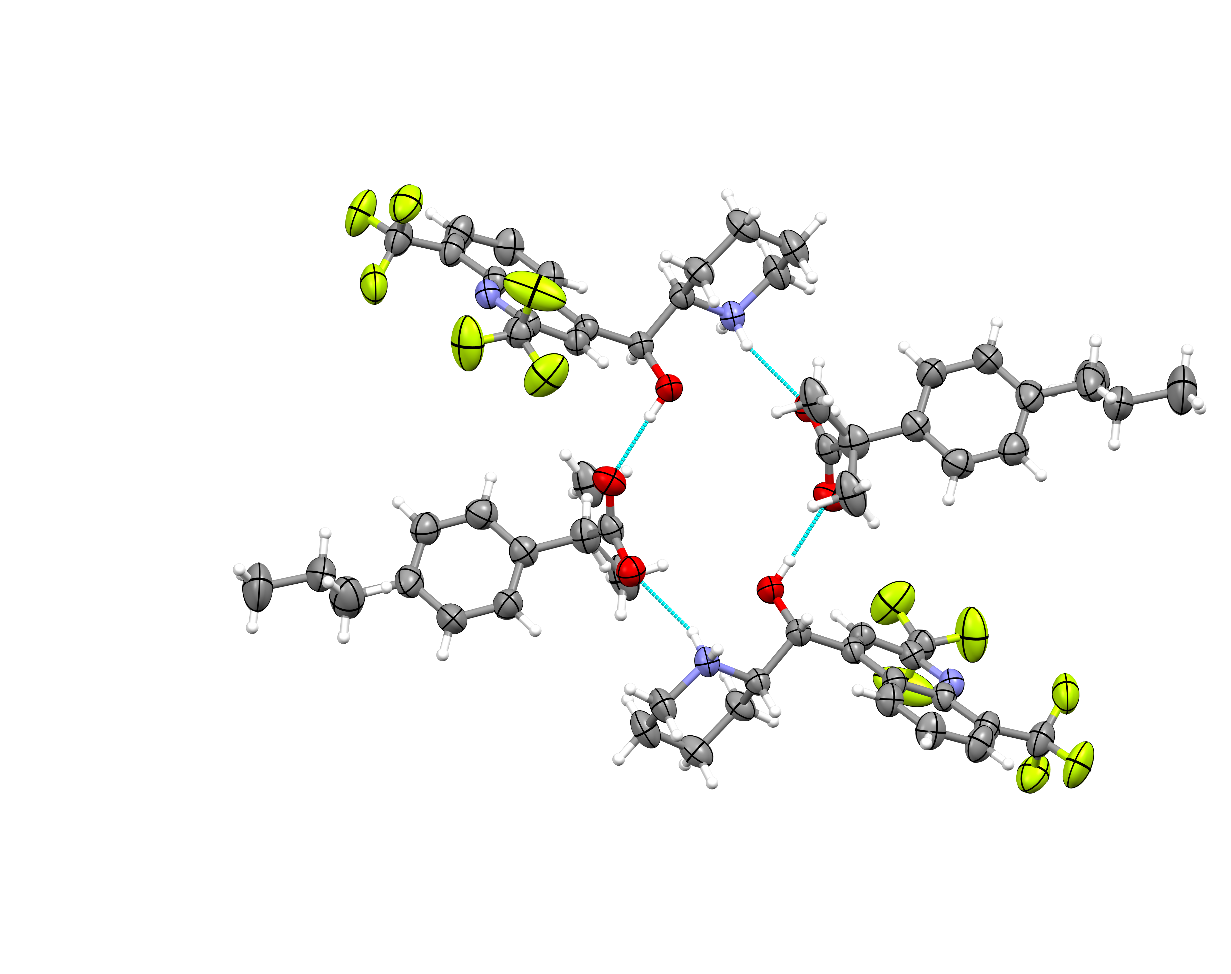


**Figure S5.** ADDSYM analyses in Platon for (a) Mf-S-Kt and (b) Mf-S-Ib. Although the *P*2/n and *P*2_1_/c space groups were proposed for Mf-S-Kt and Mf-S-Ib, respectively, these are incompatible with the presence of enantiopure anions within the crystal lattice. Such pseudo-symmetry is a frequent challenge in the characterization of double salts. Attempts to refine these structures within centrosymmetric space groups led to significant anionic disorder and failed to achieve model convergence.

| **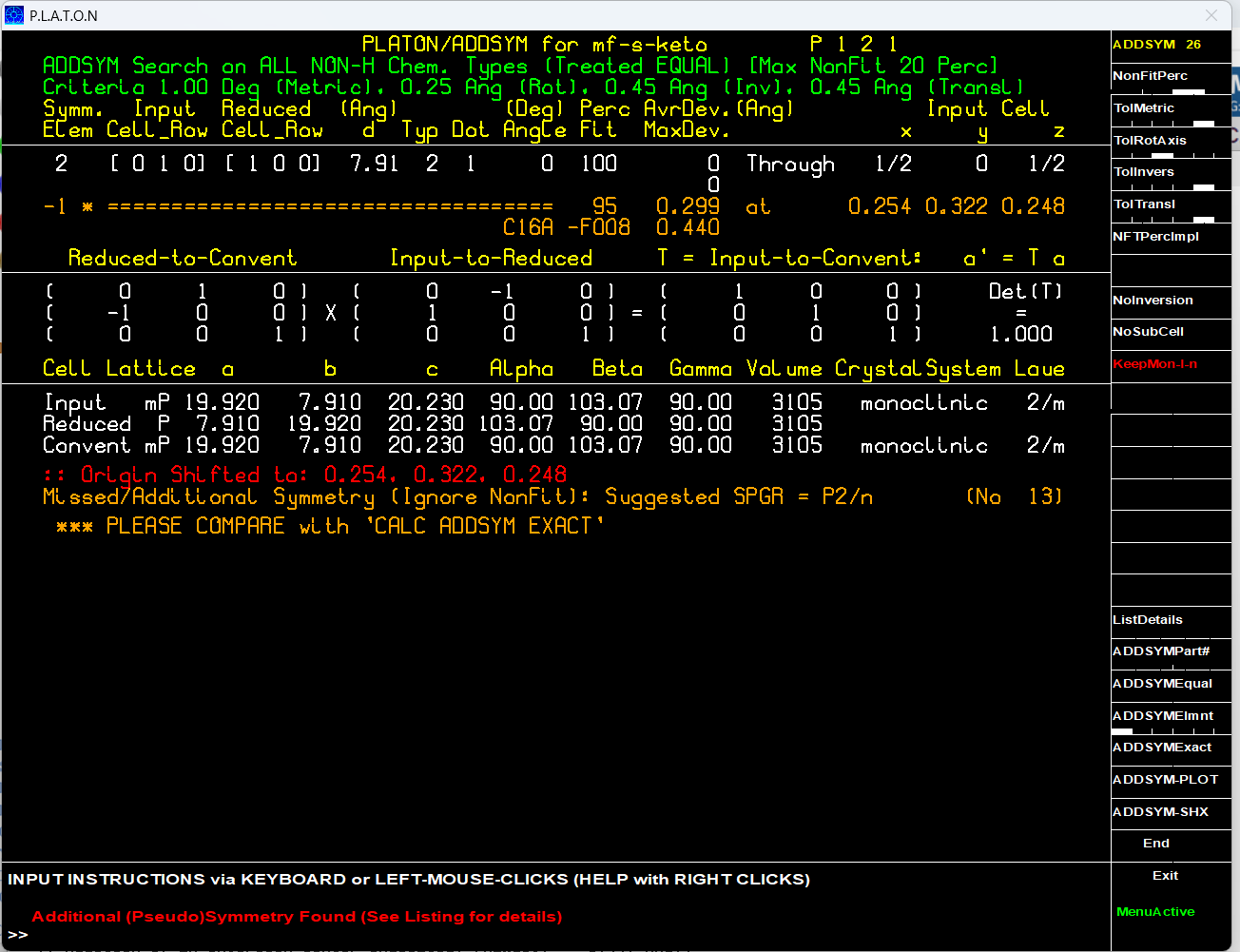** |
| --- |
| **(a)** |
| **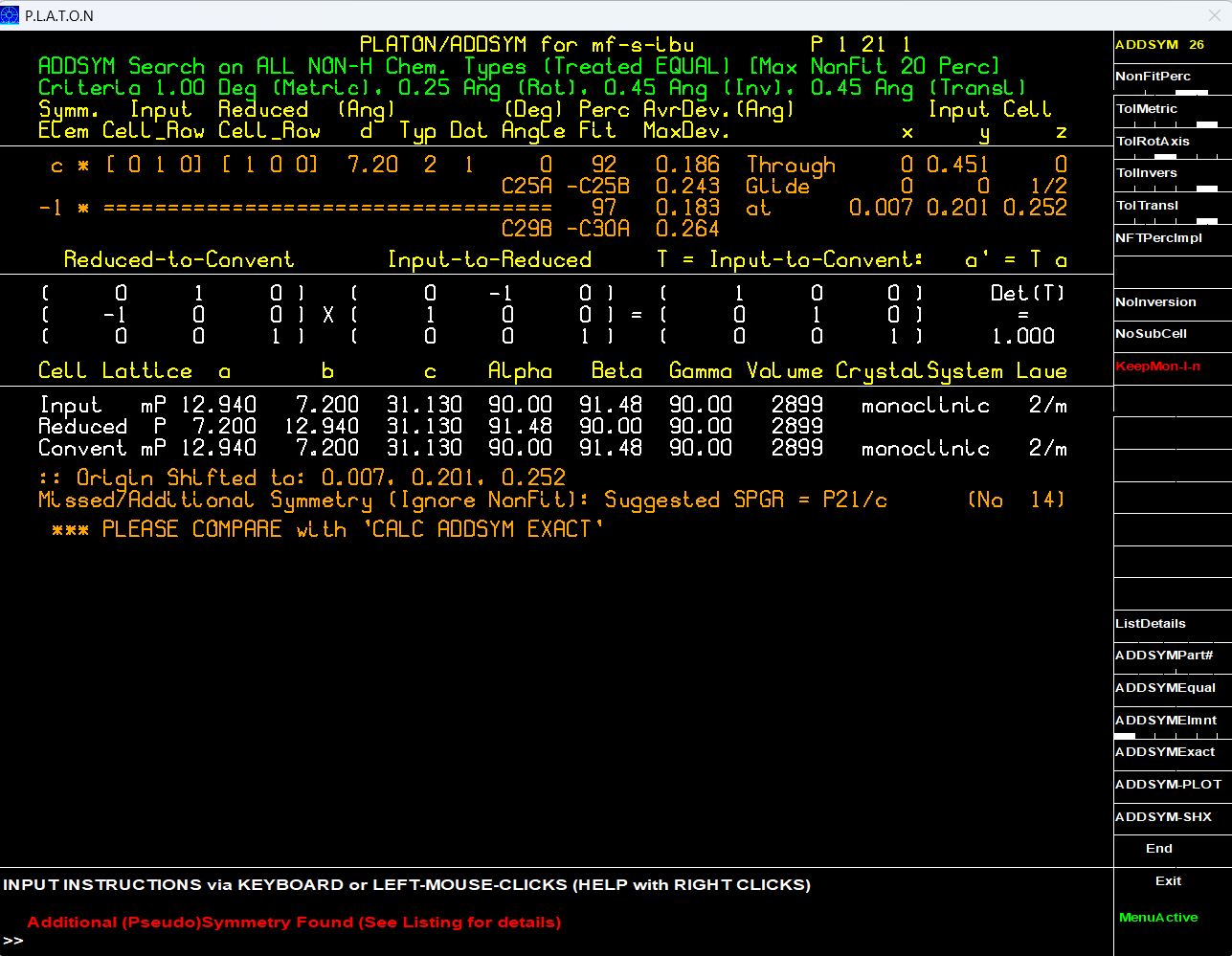** |
| **(b)** |

**Figure S6**. Overlay of the crystal packing arrangements of Mf-S-Kt and Mf-Kt.


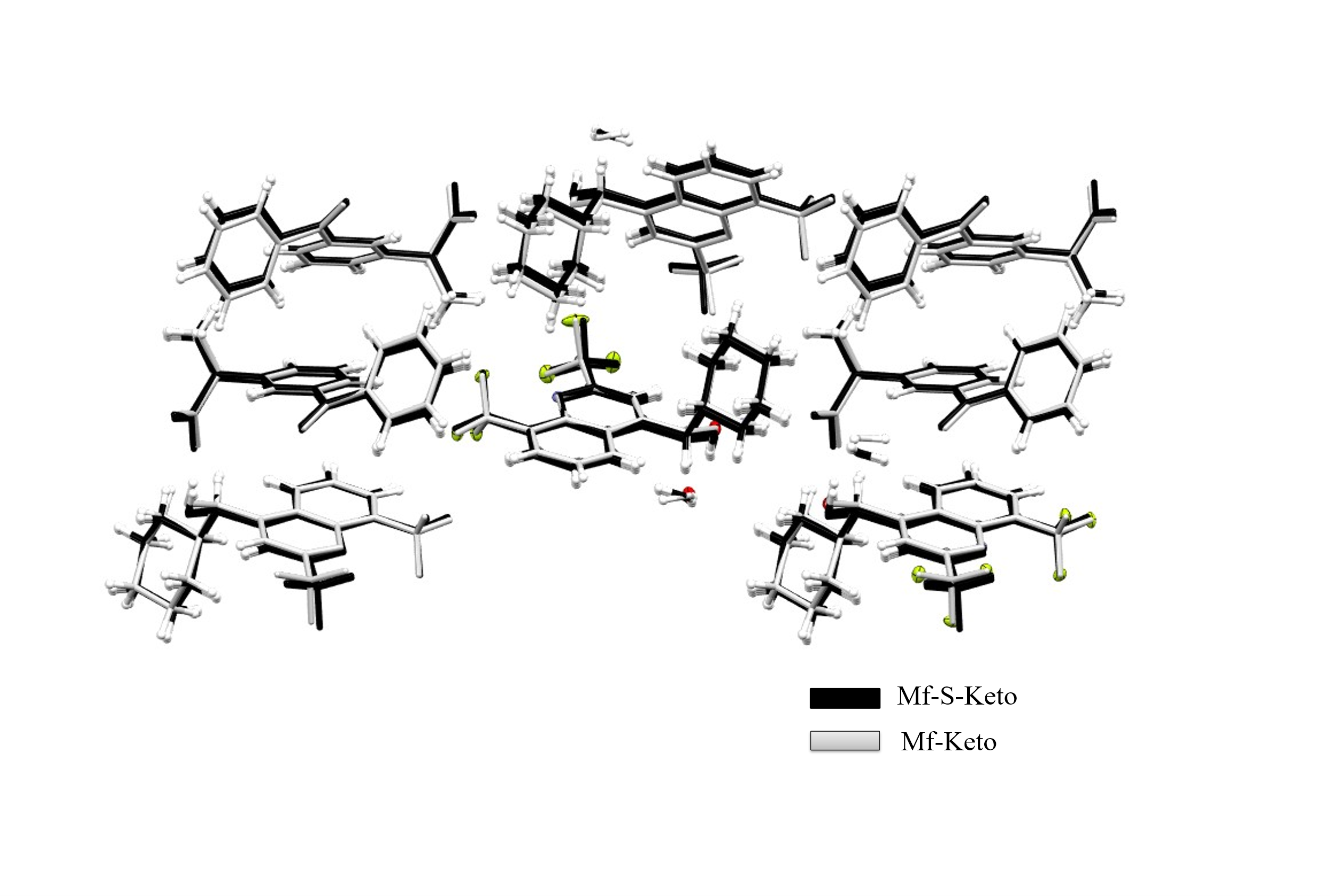


**Figure S7.** Percentage of intermolecular contact in the salts.
